# Effects of Dual-Task Versus Multicomponent Exercise Programs on Fear of Falling and Fall Risk in Institutionalized Older Adults: A Randomized Controlled Trial

**DOI:** 10.3390/healthcare14080981

**Published:** 2026-04-09

**Authors:** Daniela Pereira, Filipe Rodrigues

**Affiliations:** 1 ESECS—Polytechnic of Leiria, 2411-901 Leiria, Portugal; 1241412@my.ipleiria.pt; 2 Research Center in Sport, Health and Human Development (CIDESD), 5000-558 Vila Real, Portugal

**Keywords:** frailty, homes for the aged, accidental falls, fear, exercise, randomized controlled trial

## Abstract

**Background/Objectives**: Institutionalized aging is associated with severe physical deconditioning, a high risk of falls, and a pervasive fear of falling. Physical exercise mitigates these factors, but the comparative efficacy of different training methodologies in this specific population remains unclear. The objective of this study was to compare the impact of a multicomponent exercise program versus a dual-task (cognitive-motor) training program on reducing fall risk, decreasing the fear of falling, and improving physical performance in institutionalized older adults. **Methods**: A randomized, parallel group controlled trial involving 21 older adults residing in a nursing home (Mean age = 83.67 ± 6.17 years). Participants were allocated to either a Multicomponent Group (n = 11) or a Dual-Task Group (n = 10) for a 12-week intervention (2 sessions/week). Fall risk, fear of falling, and global physical performance were assessed at baseline and post-intervention. **Results**: No significant improvements were observed in fall risk assessment execution time for either group. The Multicomponent Group showed a significant reduction in the fear of falling (−29.1%; 95% CI [−17.27, −1.27], *p* = 0.025) and a clinically significant improvement in physical performance (+40.9%; 95% CI [1.11, 3.43], *p* < 0.001), supported by large time effects (FES-I: F(1, 19) = 4.52, η^2^p = 0.192; SPPB: F(1, 19) = 13.68, η^2^p = 0.419). The Dual-Task Group achieved no significant changes in these dimensions. Furthermore, a marginally significant time-by-group interaction was observed for physical performance, favoring the multicomponent approach (F(1, 19) = 3.83, *p* = 0.065, η^2^p = 0.168 [large effect]). **Conclusions**: Multicomponent training proved superior in improving physical performance and reducing the fear of falling. In a frail, institutionalized population, the attentional cost demanded by dual-task training appears to limit the physical and psychological benefits of exercise.

## 1. Introduction

Global demographic aging poses substantial challenges to public health, particularly regarding the maintenance of physical and psychological independence in older populations. During the aging process, systemic physiological changes occur, most notably the loss of muscle mass and strength, a phenomenon known as sarcopenia, which directly affects mobility and postural stability [1]. When analyzing the older population residing in nursing homes or long-term care facilities, the scenario is even worse. The physical performance of institutionalized older adults is frequently more compromised than that of their community-dwelling peers. Institutionalization is typically associated with lower levels of daily physical activity, a higher prevalence of chronic comorbidities, and an accelerated decline in basic motor functions, such as gait and balance [2,3]. Consequently, the frailty status of these populations demands specific interventions that address both physical strengthening and functional self-confidence [4,5].

The decline in physical performance and the impairment of the neuromuscular and sensory systems translate into a significantly increased risk of falling. Falls are not random events, but rather the result of a complex interaction between intrinsic and extrinsic risk factors [6]. Evidence demonstrates that decreased lower-limb muscle strength, combined with agility deficits, is one of the primary predictors of falls in older adults [7]. Faced with this imminent risk, prevention becomes a clinical priority, making the implementation of effective prophylactic strategies crucial [8].

Parallel to the physical risk emerges a highly disabling psychological barrier: the fear of falling. Fear of falling is defined as a persistent concern about the possibility of falling that leads individuals to avoid performing activities of daily living that they are still capable of executing [9]. This phenomenon can manifest in two distinct ways: before a fall occurs or exacerbated after a fall episode. In the absence of a fall history, fear may arise from the perception of frailty, observing peers fall, or progressive sensory deficits [10,11]. However, heightened fear of falling typically manifests in the post-fall period. Individuals who have already fallen, especially those resulting in vertebral fractures or immobilization, develop severe anxiety regarding movement [12,13]. This fear establishes a harmful vicious cycle. The concern about falling leads to a voluntary restriction of physical and social activities, which in turn accelerates physical deconditioning, muscle atrophy, and loss of balance, culminating in a real and even greater risk of experiencing new falls [14,15].

### 1.1. The Impact of Physical Exercise on Fall Risk and Fear of Falling

Intervention through physical exercise has proven to be the most potent and effective non-pharmacological tool to break this cycle. The scientific literature supports the idea that regular and properly structured physical exercise decreases the risk of falls. The manipulation of training variables, such as volume and intensity in resistance exercises, induces vital neuromuscular adaptations, improving muscle quality and physical performance [16]. Furthermore, approaches that combine different energy pathways, such as concurrent training that links strength to aerobic endurance, have been shown to be compatible and beneficial for hypertrophy and functionality in older adults [17,18]. Similarly, physical exercise has a profound and direct impact on decreasing the fear of falling. Exhaustive systematic reviews and meta-analyses confirm that exercise interventions reduce concern and fear of falling in older adults [19,20,21]. Continued practice restores the older adult’s perception of physical self-efficacy. Specific and validated programs, such as Otago, have reported significant reductions in fear of falling, promoting empowerment and mobility [22]. Additionally, interventions that integrate exercise with health education, exposure therapies, and cognitive-behavioral approaches show even more promising results in attenuating anxiety responses to movement and reintegrating older adults into their routines [23,24,25].

### 1.2. Types of Programs: Multicomponent and Dual-Task Training

To optimize functional and psychological prevention, different exercise program designs have been applied, most notably Multicomponent Training and Dual-Task Training. Multicomponent Training is characterized by the integration of different physical capacities in the same session or training cycle, usually encompassing strength, cardiovascular endurance, balance, and flexibility exercises. The objective is to provide a holistic stimulus that combats the multiplicity of deficits associated with aging [26]. By acting simultaneously on muscle strength (essential for postural reactivity) and static and dynamic balance, this program ensures that the older adult recovers the ability to stabilize their center of gravity. As a result, the risk of falling decreases due to the increase in physical robustness, and the fear of falling is reduced because the older adult gains confidence in their global motor capacities to support their own body [27]. In turn, Dual-Task Training consists of the simultaneous execution of a motor task (i.e., primary, such as walking or maintaining postural balance) and a cognitive task (i.e., secondary, such as performing mental arithmetic calculations, naming animals, or memorizing sequences). The relevance of this methodology is based on the fact that, in daily life, falls frequently occur when the older adult’s attention is divided (e.g., walking while talking or paying attention to an obstacle). Aging reduces attentional processing capacity, which impairs gait when there is cognitive interference. Dual-task training acts precisely on this limitation, inducing neuroplasticity and improving reaction time and gait stability under cognitive load. By exposing the older adult to these perturbations in a controlled environment, a direct transfer to everyday demands is promoted, further decreasing the risk of falls and attenuating the fear of falling through habituation to attention focus management [28,29].

### 1.3. Literature Gap and Study Rationale

Despite the abundance of evidence regarding the benefits of exercise, the literature presents some gaps. The vast majority of clinical trials and reviews focus on independent or community-dwelling older adults [27], leaving a scarcity of robust data regarding the institutionalized population, which possesses substantially more severe levels of motor dependence and cognitive decline. Moreover, although both multicomponent and dual-task training show isolated efficacy, there is a lack of studies directly comparing the impact of both within the same clinical trial to determine which methodology offers the best dose–response relationship in mitigating fall risk and fear of falling in a nursing home setting. The present study aims to fill these gaps by applying and comparing these two programs in a specific sample of institutionalized older adults, helping to clarify exercise prescription guidelines for this vulnerable population.

### 1.4. Objectives and Hypotheses

The primary objective of this study is to analyze and compare the impact of a multicomponent exercise program and a dual-task (cognitive-motor) training program on the reduction in fall risk and fear of falling in institutionalized older adults. To this end, the following hypotheses were established:

**Hypothesis 1 (H1).**  *The implementation of a structured exercise program, whether multicomponent or dual task, significantly decreases fall risk and fear of falling in institutionalized older adults compared to their baseline levels. This prediction is supported by robust literature demonstrating that regular physical exercise counteracts severe physical deconditioning, induces vital neuromuscular adaptations, and restores physical self-efficacy, thereby breaking the vicious cycle between frailty and the fear of movement.*

**Hypothesis 2 (H2).**  *The group subjected to the dual-task training program will show a significantly greater reduction in fall risk and fear of falling compared to the multicomponent training group, due to the specificity of the neuromotor and attentional stimulus required to resolve real-life situations. As detailed earlier, this hypothesis is grounded in the premise that dual-tasking mimics the divided attention frequently required in daily life. Therefore, we expected it to provide a more specific and transferable neuromotor and attentional stimulus to resolve real-life situations than purely physical training.*

## 2. Materials and Methods

### 2.1. Study Design and Participants

**Figure 1:**
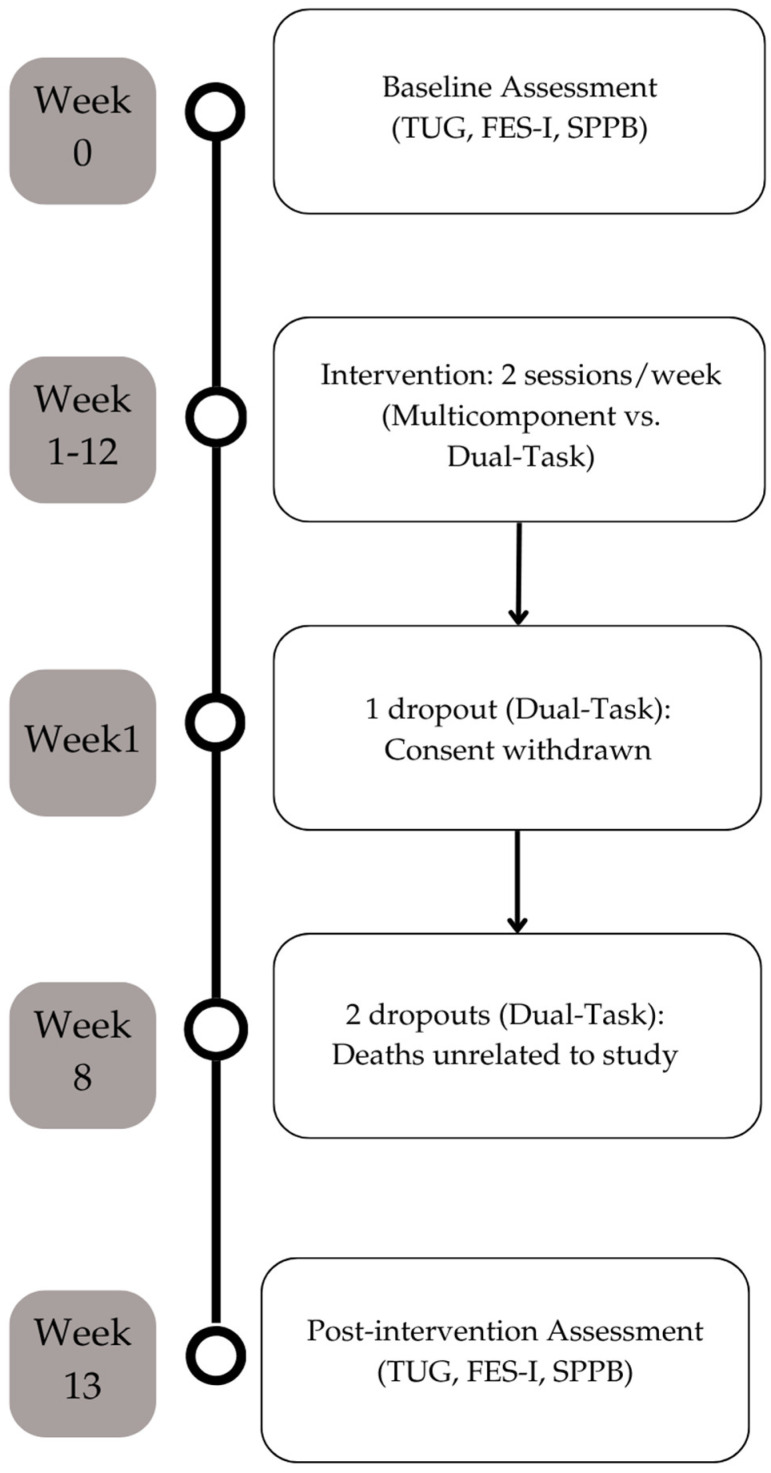
Study design and intervention timeline.

The present study is a randomized, controlled, parallel-group clinical trial with assessor blinding. The research protocol is registered on the ClinicalTrials.gov platform (registration number NCT07427225, date: 17 February 2026). The research was approved by the Ethics Committee of the Polytechnic of Leiria (approval CE/IPLEIRIA/88/2025 on 5 December 2025) and conducted in strict compliance with the Declaration of Helsinki. The conduct and reporting of this study followed the CONSORT (Consolidated Standards of Reporting Trials) guidelines for non-pharmacological trials (CONSORT checklist can be found at Table S1). All participants, or their legal representatives, signed the informed consent prior to inclusion in the study. The experimental protocol, assessment points, and the temporal distribution of participant attrition over the 12-week period are visually detailed in Figure 1.

The sample was recruited from the population residing in a nursing home in the Leiria region, Portugal. Participant selection was based on the following inclusion criteria: (i) age 65 years or older; (ii) prior institutionalization for a minimum period of 6 months; (iii) independent walking ability, regardless of the use of assistive mobility devices such as canes or walkers; (iv) medical authorization and clearance to practice physical exercise; and (v) ability to communicate and understand simple verbal instructions, reflected by the absence of severe cognitive decline. Individuals were excluded if they presented: (a) uncontrolled cardiovascular or metabolic diseases (e.g., severe heart failure, unstable angina, or myocardial infarction in the previous month); (b) severe musculoskeletal, visual, or auditory pathologies that would prevent the safe performance of exercises; and (c) a diagnosis of severe dementia and/or severe neurological disorders that would hinder adherence to the protocol. Additionally, an attendance rate of less than 75% of the total program or an absence of more than 5 consecutive sessions was established as an exclusion criterion for the Per-Protocol analysis.

Sample size was determined a priori using G*Power software (v.3.1.9.7). The calculation was based on an F-test for a repeated measures ANOVA (within-between interaction), assuming a large effect size (f = 0.50), an alpha level of 0.05, a statistical power of 0.95 (1 − β), and a correlation among repeated measures of 0.50. The calculation indicated a minimum requirement of 54 participants.

### 2.2. Data Collection Procedures

Prior to the study’s commencement, formal contact was established with the institution’s management to present the project and obtain the necessary authorization for its implementation. Upon receiving a favorable opinion, a review of the residents’ files was conducted in close collaboration with the institution’s clinical and medical team for the initial screening and identification of potential participants who met the pre-established inclusion criteria.

The older adults flagged in this initial phase were subsequently contacted individually and in person by the research team. During this approach, the study’s objectives, the nature of the assessments, and the total duration of the intervention (12 weeks of physical exercise program) were explained clearly and accessibly. The strictly voluntary nature of participation was emphasized. They were explicitly informed that they had the freedom to withdraw their participation at any time during the study without the need for justification and without this implying any penalty in the care provided by the institution. After providing all clarifications, the individuals who agreed to join the investigation signed a voluntary and informed consent form. The data collected were anonymized using an alphanumeric coding system and stored on a private server, complying with the General Data Protection Regulation.

Following the recruitment phase, baseline data (week 0) were collected. Participants were then randomly allocated to one of two groups: Control Group (Multicomponent Training) or Experimental Group (Dual-Task Training). This division was conducted using a simple randomization procedure with a 1:1 allocation ratio. The randomization sequence was generated using computer software Research Randomizer (Version 4.0) by an independent researcher not involved in the direct assessment of the participants, thereby ensuring allocation concealment. To minimize bias within the institutional setting, participants were blinded to the specific research hypotheses. They were informed that the study aimed to compare two different, yet potentially effective, exercise programs for health promotion, maintaining a sense of treatment equipoise. This strategy was intended to prevent potential social biases of equalization or demoralization that could arise if participants discussed their routines. Furthermore, all physical and psychological assessments were conducted by independent researchers who remained strictly blinded to group allocation throughout the trial, ensuring that the primary source of potential bias (the assessor) was controlled.

Assessments were conducted on the institution’s own premises, using standardized equipment. To ensure the rigor of the blinded design, the first author (D.P.) implemented the intervention sessions but performed no assessments. Conversely, the second author (F.R.), acting as the blinded assessor, conducted all motor and psychological tests. F.R. was not involved in the training sessions, was not present during the exercise periods, and remained unaware of the participants’ group allocation until all post-intervention data were recorded.

### 2.3. Instruments

Prior to administering the performance tests and specific questionnaires, sociodemographic and clinical data were collected. Using an initial structured questionnaire, information such as age, sex, marital status, education level, time of institutionalization, and medical history, including comorbidities and history of falls, was recorded. After completing this sociodemographic questionnaire, participants performed the tests and completed the respective assessment scales. All selected instruments are properly translated, adapted, and validated for the Portuguese population, ensuring the psychometric rigor, reliability, and cultural appropriateness of the collected data.

Fear of falling was assessed using the Falls Efficacy Scale-International (FES-I) [30]. This self-report scale evaluates the level of concern about the possibility of falling while performing 16 physical and social activities of daily living. The global score ranges from 16 (no concern) to 64 points (extreme concern). The Portuguese version of this instrument has demonstrated excellent psychometric properties, namely high internal consistency (Cronbach’s α = 0.978) and excellent test–retest reliability (ICC = 0.999), indicating strong construct validity and reliability for this population [30].

Fall risk and dynamic functional mobility were assessed using the Timed Up and Go (TUG) test [31]. This is a highly clinically applicable test with well-established normative values for the older adult population in Portugal [32]. The test records the time (in seconds) it takes a participant to rise from a chair, walk a distance of three meters, turn around, walk back, and sit down again. In this population, the TUG has demonstrated excellent relative reliability, with an ICC of 0.98 (95% CI: 0.97–0.99), and high absolute reliability with a coefficient of variation of 2.2% [32].

Physical performance was evaluated using the Short Physical Performance Battery (SPPB), an instrument adapted and validated for the national clinical context [33]. The battery provides a composite score (from 0 to 12 points) that summarizes performance in three functional domains: (a) static balance (ability to maintain side-by-side, semi-tandem, and tandem stances), (b) gait speed (four-meter walk), and (c) functional lower-body strength (measured by the time required to stand up and sit down from a chair five consecutive times). The SPPB is a reliable instrument for assessing lower extremity function, with an ICC of 0.88 to 0.92 reported in large-scale studies [33]. It has also demonstrated strong predictive validity for nursing home admission and mortality, with a clear dose–response relationship between lower scores and increased risk of disability, with Hazard Ratio = 4.39 for the lowest scores [33].

Additionally, program adherence was objectively monitored and calculated by dividing the total number of sessions attended by the participant by the total number of stipulated sessions (24 sessions over the entire intervention), with the result expressed as a percentage.

### 2.4. Intervention

The intervention lasted 12 weeks, with a frequency of 2 weekly sessions and a duration of 45 to 60 min per session for both groups. Sessions were supervised by exercise professionals and structured into four sequential phases: (i) warm-up (10–15 min) focused on joint mobility and low-intensity aerobic stimulation; (ii) strength training (15–20 min) targeting major muscle groups using body weight, dumbbells, and resistance bands; (iii) balance and agility training (10–15 min) involving static and dynamic challenges; and (iv) cool-down (5–10 min) based on stretching and relaxation exercises. To accommodate the group-based nature of the sessions and the diverse functional levels of institutionalized participants, exercise volume was controlled via time under tension. Instead of fixed repetitions, participants were instructed to perform controlled movements for 45 to 60 s per set, with a 1:1 rest-to-work ratio. This time-based approach ensured that all individuals remained under physical stimulus for the same duration, regardless of their individual execution speed, preventing premature task completion or overexertion. Training intensity was maintained at a light to moderate level, individually monitored using the Talk Test [34] and the Borg Rating of Perceived Exertion scale to ensure safety and physiological progression. The use of the Talk Test as a monitoring tool is supported by its established validation against the Borg RPE scale, with previous research indicating that RPE values are significantly associated with Talk Test stages, showing a strong correlation (r = 0.71) in healthy and clinical populations [34]. The use of the Talk Test ensured that participants exercised at an intensity where they could comfortably maintain a conversation, preventing overexertion.

In the Control Group (Multicomponent Training), participants performed the aforementioned physical exercise program in the form of single tasks, focusing their attention exclusively on motor and postural execution.

In the Experimental Group (Dual-Task Training), participants performed the exact same physical protocol regarding volume, frequency, and intensity, with the substantial difference being the simultaneous addition of cognitive demands. The goal was to induce cognitive-motor interference, mimicking everyday constraints. While executing the motor exercises, participants were asked to concurrently perform continuous cognitive tasks, such as verbal fluency exercises (e.g., naming animals or colors), mental arithmetic calculations (e.g., serial subtractions), and working memory recall exercises.

### 2.5. Statistical Analysis

Data processing and analysis were performed using IBM SPSS Statistics software, version 30.0 (IBM Corp., Armonk, NY, USA). Descriptive data are presented as mean ± standard deviation for continuous variables. Additionally, the percentage change (Δ) between the pre- and post-intervention moments was calculated for each group using the formula: Δ = [(Post-test mean − Pre-test mean)/Pre-test mean] × 100. The normality of data distribution was assessed using the Shapiro–Wilk test. During preliminary exploratory analysis, an extreme outlier was identified in the functional mobility variable (value > 3 standard deviations from the mean), which was excluded from the final analysis to ensure compliance with the assumptions for parametric tests. Homogeneity of variances was verified using Levene’s test, and homogeneity of covariance matrices using Box’s M test (detailed results are provided in Appendix A, Table A1). To analyze the effects of the intervention programs, a 2 × 2 mixed-design ANOVA was used, considering time (Pre vs. Post-intervention) as the within-subject factor and group (Multicomponent vs. Dual-Task) as the between-subjects factor. Whenever significant interactions or relevant main effects were observed, multiple comparisons with Bonferroni adjustment were performed to identify specific differences between time points and groups. This parametric procedure was justified by the normality of the data distribution, as confirmed by the Shapiro–Wilk test (detailed *p*-values for all variables are provided in Appendix A, Table A1). For the fear of falling (FES-I) variable, where the homogeneity assumption was violated at post-intervention (*p* < 0.001), Pillai’s Trace was reported to ensure statistical robustness. Effect size was estimated using partial eta squared (η^2^p) and interpreted according to Cohen’s suggested reference values: η^2^p = 0.01 (small effect), η^2^p = 0.06 (medium effect), and η^2^p > 0.14 (large effect) [35]. All statistical tests were two-tailed, with the significance level set at *p* < 0.05.

## 3. Results

**Figure 2:**
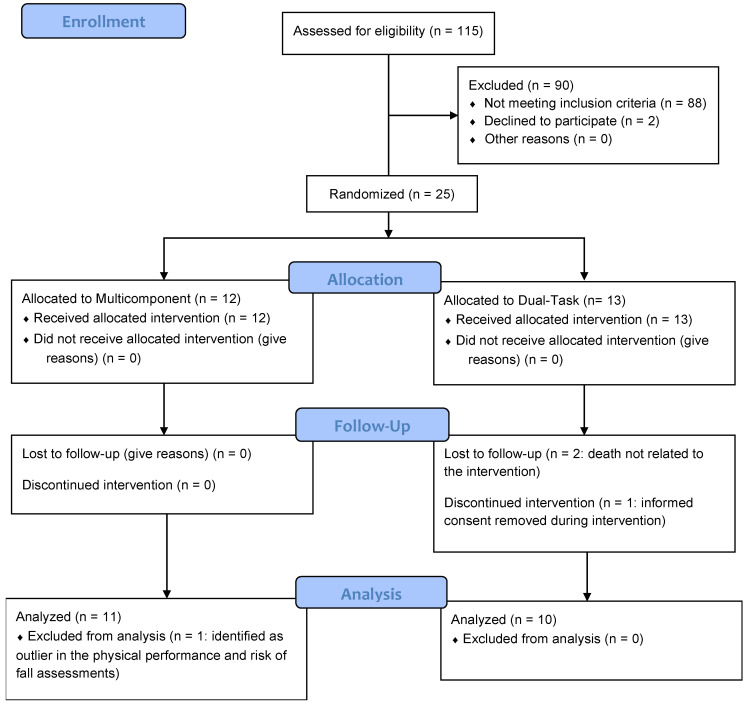
CONSORT flow diagram.

From an initial flow of 115 participants assessed for eligibility, a total of 25 older adults met the inclusion criteria and were randomized to one of the two experimental groups: Multicomponent Group (n = 12) and Dual-Task Group (n = 13). During the intervention phase, there were three dropouts in the Dual-Task Group: two participants passed away due to causes unrelated to the study, and one participant withdrew consent after the first week. In the Multicomponent Group, all participants completed the intervention protocol. However, one participant was excluded from the final analysis a posteriori after being identified as an extreme outlier in the physical performance tests, compromising statistical assumptions. Consequently, the final sample included in the statistical analysis consisted of 21 participants: 11 in the Multicomponent Group and 10 in the Dual-Task Group. The detailed flow of participants is illustrated in Figure 2.

The sociodemographic and clinical characteristics of the participants at baseline are summarized in Table 1. The final sample consisted of 21 institutionalized older adults (Mean age = 83.67 ± 6.17 years), mostly female (66.7%) and widowed (71.4%). There were no statistically significant differences between the groups (Multicomponent vs. Dual-Task) at baseline regarding age, sex, marital status, or education level (*p* > 0.05). Regarding body composition, although the Dual-Task Group presented a numerically higher BMI with a large sample effect size (Cohen’s d = 0.87), the 95% confidence interval crossed zero [−7.97, 0.21] and the difference did not reach statistical significance (*p* = 0.062), indicating baseline comparability between the groups. 95% confidence intervals and effect sizes are shown in Table 1.

The effects of the interventions on the outcome variables (TUG, FES-I, and SPPB) were analyzed using repeated measures ANOVA. All assumptions of normality and homogeneity of variances were met, except for the FES-I at the post-intervention moment (Levene’s Test *p* < 0.001), for which Pillai’s Trace is reported. Descriptive and inferential data are summarized in Table 2. Regarding functional mobility (TUG), no significant main effects or time-by-group interactions were observed (*p* > 0.05). However, significant findings were noted in both the psychological and overall physical performance domains. For the fear of falling (FES-I), a significant main effect of time was observed (F(1, 19) = 4.52, *p* = 0.047, η^2^p = 0.192). Pairwise comparisons confirmed that only the Multicomponent Group achieved a significant reduction in their fear of falling (*p* = 0.025). Finally, overall physical performance (SPPB) exhibited a large and significant main effect of time (F(1, 19) = 13.68, *p* = 0.002, η^2^p = 0.419), alongside a marginally significant time-by-group interaction favoring the multicomponent approach (F(1, 19) = 3.83, *p* = 0.065, η^2^p = 0.168 [large effect]). Specifically, the Multicomponent Group demonstrated a clinically relevant intra-group improvement of 2.27 points (*p* < 0.001), whereas the Dual-Task Group showed only a modest, non-significant increase.

## 4. Discussion

The present study aimed to analyze and compare the impact of a multicomponent exercise program and a dual-task training program on the reduction in fall risk and fear of falling in a sample of institutionalized older adults. Based on the obtained results, the first formulated hypothesis (H1) was only partially confirmed. While, on one hand, global improvements were observed in physical performance and in the reduction in fear of falling over time, on the other hand, the fall risk measured by the dynamic functional mobility test did not undergo statistically significant changes in either group. Additionally, the second hypothesis (H2) was entirely rejected: the group subjected to dual-task training did not show a greater reduction in fall risk and fear of falling. On the contrary, the multicomponent training group demonstrated more robust intragroup results, with significant decreases in fear of falling and clinically relevant improvements in physical performance, evidence of a superior dose–response relationship for this specific population.

The lack of significant improvements in dynamic balance measured by the Timed Up and Go in both interventions warrants careful reflection. The literature has demonstrated that gait and agility are critical determinants for fall prevention [7,8]. However, the institutionalized population presents a picture of motor frailty and physical deconditioning that is substantially more severe and chronic than that of the community-dwelling population [1,2,3]. It is plausible that a 12-week period, although sufficient to induce basic neuromuscular adaptations, did not have the necessary duration or cumulative volume for these adaptations to translate into the biomechanical complexity required by dynamic balance and changes in direction inherent to the TUG. Reversing the functional risk of falls in a population with this level of institutionalization may require more prolonged interventions to break the inertia of severe inactivity [5,6].

On the other hand, the results regarding overall physical performance reveal a fundamental divergence between the two methodological approaches, which helps to explain the rejection of our second hypothesis. The multicomponent group achieved an increase of over 2 points in overall physical performance. From a clinical standpoint, this improvement may be impactful. It exceeds the established minimal clinically important difference of 1 point for the SPPB in older adults. In a practical, institutionalized setting, a 2-point shift can reclassify a patient from a state of severe functional limitation to moderate frailty. This may translate into an enhanced ability to perform basic activities of daily living, such as safely transferring from a bed to a chair or walking to the dining hall independently, thereby preserving the individual’s residual autonomy. The dual-task group, however, obtained only marginal gains. The justification for this discrepancy most likely lies in the principle of cognitive-motor interference and the dilution of training stimulus intensity. During the execution of exercises, adequate manipulation of volume and intensity is a non-negotiable factor for obtaining adaptations in muscle strength and quality [16]. By performing only simple tasks, the participants in the multicomponent group were able to channel all their attention and neuromuscular resources towards motor execution, optimizing the recruitment of motor units [26,27].

Conversely, in the dual-task group, the simultaneous introduction of cognitive challenges appears to have generated a high attentional cost. Although dual-task training is frequently highlighted as highly effective in the literature [28,29], a direct comparison with recent trials clarifies this discrepancy. For instance, Chen et al. [36] demonstrated that a dual-task intervention yielded greater improvements in functional fitness than exercise alone, but their sample consisted of more robust, community-dwelling older adults. Conversely, when observing institutionalized populations, our findings align perfectly with the recent AgeingOn Dual-Task trial by Rezola-Pardo et al. [37], which concluded that simple multicomponent training was superior to dual-tasking for reducing fall rates and mitigating fall risk in nursing home residents. In these institutionalized populations, where cognitive processing capacity and motor reserve are already diminished, the requirement to perform calculations or evoke memories during a squat or balance exercise can force the brain to prioritize the cognitive task to the detriment of the motor one [11]. As a consequence, range of motion may decrease, execution speed slows down, and the mechanical intensity of the exercise drops to suboptimal levels, failing to generate sufficient stimulus for hypertrophy or functional strength gains, as evidenced by the poor physical performance test results in this group. This interference corroborates the perspective that the compatibility between different demands in the same training must be carefully managed in vulnerable populations [17,18].

This phenomenon of interference and overload also clearly explains the results found in the psychological dimension, namely the fear of falling. Fear of falling appears to be a highly limiting factor that induces activity restriction and perpetuates atrophy and deconditioning [9,10,14,15]. Our study demonstrated that multicomponent training reduced this fear by almost 30%, a reduction of over 9 points in the FES-I score. Clinically, this is a seems to be a profound change. It signifies a transition from extreme avoidance of movement to a possible confident re-engagement in the institutional routine. Overcoming this psychological barrier may be critical to preventing the rapid, downward spiral of sarcopenia and social isolation that typically follows fear-induced immobility, a remarkable impact that fully aligns with strong evidence associating exercise with improved self-efficacy [19,20,21]. Interventions of a purely physical and focused nature, such as the Otago program, have shown similar success by restoring older adults’ perception of control over their own bodies [4,22]. By focusing exclusively on movement, the older adult experiences an environment of safe exposure to risk, gradually gaining confidence [24,25].

Conversely, in the dual-task group, the simultaneous introduction of cognitive challenges appears to have generated a high attentional cost, blunting the physical and psychological adaptations. The efficacy of dual-task training is widely debated in the literature and appears to be heavily dependent on the participants’ baseline cognitive and motor reserves. By analyzing recent studies across the lifespan, a clear continuum of cognitive-motor interference emerges. In young, healthy individuals, acute dual-task interventions are easily tolerated and effectively stimulate executive function [38]. Moving along the aging spectrum, Chen et al. [36] demonstrated that in robust, community-dwelling older adults, a dual-task multicomponent intervention yielded significantly greater improvements in both functional fitness and cognitive performance than exercise alone. In these independent populations [28,29,37], the superimposed cognitive challenge provides an optimal stimulus that mimics real-life constraints, promoting neuroplasticity without compromising mechanical execution. However, our findings suggest that this paradigm does not directly translate to the institutionalized geriatric setting. In frail populations, where cognitive processing capacity and motor reserve are already critically diminished, the requirement to perform calculations or evoke memories during a squat or balance exercise acts as an insurmountable barrier rather than a beneficial stimulus. Forced to divide their attention, frail older adults naturally prioritize the cognitive task to the detriment of motor execution [11], resulting in decreased range of motion, slower movement, and suboptimal mechanical intensity. Our clinical observations are supported by the recent AgeingOn Dual-Task trial by Rezola-Pardo et al. [37], which compared multicomponent and dual-task training in long-term nursing home residents. They found that the dual-task group experienced a 3.8 times greater risk of falling compared to the multicomponent group, concluding that simultaneous cognitive demands likely compromised exercise execution and postural safety in this vulnerable sample [37].

This phenomenon of cognitive-motor interference explains the poor physical performance and the lack of improvement in the fear of falling observed in our dual-task group. While the multicomponent group focused their neuromuscular resources towards restoring physical capacity, leading to a 30% reduction in fear of falling, the dual-task group struggled with the sensation of instability. If an older adult who already possesses a fear of movement is forced to divide their attention, the perception of insecurity may increase, negating the empowerment effect that exercise should provide. The inability to focus attention on postural stabilization meant that the exercise may not have act as an effective exposure therapy [23], keeping low confidence levels regarding the possibility of falling almost unchanged.

The analysis of these results suggests an important paradigm shift in the approach to institutionalized older adults. The widespread belief that dual-task training is invariably superior because it mimics real-life constraints must be viewed in light of the individual’s initial condition. In an institutionalized population, characterized by advanced frailty, simple multicomponent training proves to be the primary intervention of choice. This method allows focusing the entirety of attentional resources on the recovery of basic motor capacities (i.e., strength and static balance), resulting in tangible functional improvements that translate into a drastic recovery of psychological confidence and mitigation of the fear of falling. The introduction of cognitive tasks superimposed on movement should, therefore, be viewed not as a starting point, but as an advanced progression phase, to be implemented only when the older adult has recovered sufficient functional strength and self-efficacy reserve to withstand the complexity of the stimulus without compromising mechanical intensity and perceived safety.

### Limitations of the Study

The interpretation of the present study’s results must be conducted with due caution, taking into consideration some methodological and contextual limitations inherent to research with institutionalized geriatric populations. Firstly, and most evidently, the small size of the final sample (n = 21) is highlighted. Although the a priori sample calculation estimated the need for 54 participants to ensure adequate statistical power, the strict application of eligibility criteria—necessary to guarantee intervention safety—combined with mortality and losses to follow-up typical of this frail age group, limited the number of participants. This sample constraint decreases the statistical power of the analysis, increasing the risk of Type II errors. That is, the inability to detect statistically significant differences between groups when these may actually exist at the clinical level. Consequently, the generalization of the conclusions to the broader population of institutionalized older adults should be done cautiously.

Secondly, the duration of the intervention constitutes a limiting factor. Although this period is documented in the literature as sufficient to observe initial neural adaptations, such as improved self-efficacy and basic motor unit recruitment, it may prove too short to induce structural changes or complex neuromotor adaptations. Variables requiring high dynamic postural control and agility, such as execution time in the Timed Up and Go test, may need a longer exposure period for habituation to the stimulus to translate into a measurable improvement in dynamic balance in this population.

Thirdly, the absence of a passive control group, namely a group without any exercise intervention, is noted. The non-inclusion of this group was a deliberate and ethically grounded decision, aligned with international recommendations that advocate access to physical exercise as a right and a pressing need for institutionalized older adults. However, from a strictly experimental standpoint, this absence prevents the quantification of the natural functional decline that would occur in those individuals over three months. Thus, the study focuses on the comparative efficacy between the multicomponent approach and dual-task, not isolating the absolute benefit of exercise versus inactivity. Additionally, it is important to mention the difficulty in controlling external variables. Despite participants sharing the routine of the same institution, factors with a direct impact on physical condition were not objectively monitored. Namely, individual nutritional intake and spontaneous physical activity levels outside the training sessions were not controlled, which may introduce bias in understanding each individual’s energy expenditure and recovery.

Lastly, regarding the dual-task intervention, the lack of sample stratification based on a detailed pre-intervention neuropsychological assessment limits the interpretation of the cognitive-motor cost. Given the obtained results, future clinical trials should integrate specific cognitive assessments to identify potential cognitive reserve thresholds, beyond which the introduction of a second task ceases to be a beneficial stimulus and becomes a barrier to motor performance. Furthermore, while severe neurological conditions were strictly excluded to ensure safety, we did not filter out participants with mild cognitive impairments, early-stage neurological diseases (e.g., mild stroke history), or polypharmacy. Given the high prevalence of these clinical characteristics in nursing home residents, they inherently increase sample heterogeneity and may have acted as uncontrolled confounding variables. Future research should aim to control specific pharmacological profiles. Despite these limitations, the study fulfills its purpose of providing pragmatic data and reflecting on the clinical reality of nursing homes, emphasizing that the complexity of exercise prescription must invariably be adjusted to the functional and psychological reserve of the person executing it.

## 5. Conclusions

The present study demonstrates that, in a population of institutionalized older adults with advanced frailty, an isolated multicomponent physical exercise program proves more effective than dual-task training in improving overall physical performance and mitigating the fear of falling. The lack of superior results in the dual-task group suggests that cognitive-motor interference, potentially caused by the simultaneous demand for attention to physical and mental tasks, acts as a barrier to performance, limiting the intensity of the mechanical stimulus and preventing the gain of self-efficacy necessary to reduce anxiety toward movement. Thus, in an institutionalized context, exercise prescription in the initiation phases of a physical exercise program should prioritize simple multicomponent interventions, focused exclusively on motor optimization, reserve the superposition of cognitive tasks for more advanced rehabilitation phases, when the older adult already possesses sufficient functional reserving and confidence to tolerate the attentional cost without compromising their safety.

## Figures and Tables

**Table 1:** Sociodemographic and clinical characteristics of the sample at baseline.

Variable	Total (n = 21)	Multi (n = 11)	Dual-Task (n = 10)	95% CI of Diff.	d	*p*-Value
Age (years) Mean ± SD	83.67 ± 6.17	84.91 ± 2.66	82.30 ± 8.53	−3.61, 8.83	0.42	0.346
Sex n (%)				—	—	0.659
Female	14 (66.7%)	8 (72.7%)	6 (60.0%)			
Male	7 (33.3%)	3 (27.3%)	4 (40.0%)			
BMI (kg/m^2^) Mean ± SD	25.58 ± 4.79	23.73 ± 4.09	27.61 ± 4.87	−7.97, 0.21	−0.87	0.062
Time Institutionalized (years)	1.89 ± 1.47	1.55 ± 1.33	2.27 ± 1.60	−2.05, 0.62	−0.49	0.272
Marital Status n (%)				—	—	0.186
Married	3 (14.3%)	3 (27.3%)	0 (0.0%)			
Single	3 (14.3%)	1 (9.1%)	2 (20.0%)			
Widowed	15 (71.4%)	7 (63.6%)	8 (80.0%)			
Education Level n (%)				—	—	1.000
No education (0 years)	1 (4.8%)	0 (0.0%)	1 (10.0%)			
3rd Grade	2 (9.5%)	2 (18.2%)	0 (0.0%)			
4th Grade	13 (61.9%)	6 (54.5%)	7 (70.0%)			
6th Grade	2 (9.5%)	1 (9.1%)	1 (10.0%)			
7th Grade	1 (4.8%)	1 (9.1%)	0 (0.0%)			
8th Grade	1 (4.8%)	1 (9.1%)	0 (0.0%)			
Higher Education	1 (4.8%)	0 (0.0%)	1 (10.0%)			

Notes: SD = Standard Deviation; BMI = Body Mass Index; 95% CI of Diff. = 95% Confidence Interval of the Mean Difference; Effect Size (d) = Cohen’s d. The *p*-value refers to the Independent Samples t-Test (continuous variables) or Fisher’s Exact Test (categorical variables). Dashed lines (—) indicate metrics not applicable to categorical data.

**Table 2:** Intra- and between-group comparisons for the variables under study.

Variable	Group	Pre (M ± SD)	Post (M ± SD)	Mean Diff. (95% CI)	*p*	ANOVA Results (F; *p*; η^2^p)
TUG	Multi	15.85 ± 3.79	15.21 ± 3.85	−0.64 [−2.95, 1.67]	0.569	Time: F(1, 19) = 1.15; *p* = 0.296; η^2^p = 0.057
	Dual-Task	17.15 ± 4.44	16.07 ± 4.50	−1.08 [−3.51, 1.35]	0.363	Group: F(1, 19) = 0.44; *p* = 0.516; η^2^p = 0.023
						Time × Group: F(1, 19) = 0.08; *p* = 0.786; η^2^p = 0.004
FES-I	Multi	31.91 ± 10.63	22.64 ± 3.01	−9.27 [−17.27, −1.27]	0.025	Time: F(1, 19) = 4.52; *p* = 0.047; η^2^p = 0.192
	Dual-Task	32.10 ± 8.66	29.60 ± 9.44	−2.50 [−10.89, 5.89]	0.540	Group: F(1, 19) = 2.18; *p* = 0.157; η^2^p = 0.103
						Time × Group: F(1, 19) = 1.50; *p* = 0.236; η^2^p = 0.192
SPPB	Multi	5.55 ± 1.29	7.82 ± 1.72	2.27 [1.11, 3.43]	<0.001	Time: F(1, 19) = 13.68; *p* = 0.002; η^2^p = 0.419
	Dual-Task	5.30 ± 1.16	6.00 ± 2.31	0.70 [−0.52, 1.92]	0.244	Group: F(1, 19) = 2.85; *p* = 0.108; η^2^p = 0.130
						Time × Group: F(1, 19) = 3.83; *p* = 0.065; η^2^p = 0.168

Notes: M = Mean; SD = Standard Deviation; Mean Diff. = Mean difference from Pre to Post (Bonferroni adjusted); 95% CI = 95% Confidence Interval of the intra-group difference; η^2^p = Partial Eta Squared.

## Data Availability

The data presented in this study is available on request from the corresponding author. The data is not publicly available due to privacy and ethical restrictions regarding participant anonymity.

## Supplementary Materials

The following supporting information can be downloaded at: https://www.mdpi.com/article/10.3390/healthcare14080981/s1, Table S1: CONSORT Checklist.

## Appendix A

**Table A1:** Results of Statistical Assumption Tests for Normality and Homogeneity.

Variable	Shapiro–Wilk (Normality, *p*)	Levene’s Test (Variances, *p*)	Box’s M (Covariances, *p*)
TUG	Multi: 0.725 (Pre)/0.380 (Post) Dual: 0.992 (Pre)/0.226 (Post)	0.591 (Pre) 0.681 (Post)	*p* = 0.761
FES-I	Multi: 0.185 (Pre)/0.208 (Post) Dual: 0.598 (Pre)/0.269 (Post)	0.411 (Pre) <0.001 (Post)	*p* = 0.009
SPPB	Multi: 0.019 (Pre)/0.182 (Post) Dual: 0.328 (Pre)/0.034 (Post)	0.356 (Pre) 0.132 (Post)	*p* = 0.709
